# Handwriting identification and verification using artificial intelligence-assisted textural features

**DOI:** 10.1038/s41598-023-48789-9

**Published:** 2023-12-08

**Authors:** Heng Zhao, Huihui Li

**Affiliations:** College of Infommation Engineering and Artificial Inteligence, Zhengzhou Vocational University of Information and Technology, Zhengzhou, 450046 China

**Keywords:** Engineering, Mathematics and computing

## Abstract

Intelligent process control and automation systems require verification authentication through digital or handwritten signatures. Digital copies of handwritten signatures have different pixel intensities and spatial variations due to the factors of the surface, writing object, etc. On the verge of this fluctuating drawback for control systems, this manuscript introduces a Spatial Variation-dependent Verification (SVV) scheme using textural features (TF). The handwritten and digital signatures are first verified for their pixel intensities for identification point detection. This identification point varies with the signature’s pattern, region, and texture. The identified point is spatially mapped with the digital signature for verifying the textural feature matching. The textural features are extracted between two successive identification points to prevent cumulative false positives. A convolution neural network aids this process for layered analysis. The first layer is responsible for generating new identification points, and the second layer is responsible for selecting the maximum matching feature for varying intensity. This is non-recurrent for the different textures exhibited as the false factor cuts down the iterated verification. Therefore, the maximum matching features are used for verifying the signatures without high false positives. The proposed scheme’s performance is verified using accuracy, precision, texture detection, false positives, and verification time.

## Introduction

Handwriting verification or recognition process is mostly used in various fields. Handwriting verification ensures the user’s security and the organization’s safety from unknown attackers^1^. Handwriting verification is complicated in every application that provides feasible information to further security policies^2^. Various handwriting verification methods are used in the control system that first captures the content’s core or sequence^3^. Multi-semantic fusion method is widely used in handwriting verification and prediction processes. The semantic method detects spatial and temporal features of handwriting that produce optimal data for the verification process^4,5^.

Handwriting identification is the process of determining the identity of a handwritten document’s author. Given the major role of handwriting features in the writer’s classification process, the classifier accuracy is very sensitive to the weight given to each writer’s score in the feature set. This communication aims to understand better how feature selection and weight play into the handwriting recognition challenge. There are three main reasons for the problems that are occurring: (i) Handwriting recognition is mostly based on subjective experience, with small help from objective statistical methods; (ii) there are not any unified, all-encompassing evaluation standards; and (iii) experts need to get better at their tasks.

Texture feature-based handwriting signature verification methods are commonly used in various systems^6^. Texture features enhance the signature verification process’s accuracy, and maximizes the systems’ performance and effectiveness levels^7^. The feature extraction technique identifies the signature’s texture features and handwriting^8^. The support vector machine (SVM) model is used to verify handwriting signatures presented in the database and detects the important texture features^9^. Local binary patterns (LBP) and greyscale levels of signatures are verified based on certain functions^10^.

Artificial intelligence (AI) algorithms are used in handwriting signature verification to improve accuracy and reduce workload in the verification process^11^. The artificial neural network (ANN) approach is mostly used in signature verification systems^12^. Slops and patterns provide necessary information for the verification process that reduces latency in both classification and Identification^13^. A deep CNN is used for handwriting signature verification improving the systems’ performance and efficiency^14,15^.

The main objective of the paper is:

- To design the Spatial Variation-dependent Verification (SVV) scheme using textural features (TF) for handwriting identification and verification in intelligent process control and automation systems.
- Extracting textural features using a convolutional neural network for identifying points to prevent cumulative false positives.
- The experimental results have been executed, and the proposed scheme attains high accuracy, precision, texture detection, false positives, and verification time compared to existing methods.

## Related works

Li et al.^16^ designed an adversarial variation Network (AVN) model for handwritten signature verification. AVN detects the effective features that are presented in given data. The proposed AVN model maximizes verification accuracy by 94%, enhancing the systems’ performance and feasibility. However, the performance reduction speed of the technique is much slower than the baseline technique, particularly when the variation is not enormous.

Ma et al.^17^ introduced a transformer deep learning model for sequence learning is a quantitative approach that analyzed the tremor symptoms of signatures. The introduced model improves efficiency by 97.8% and an accuracy ratio of 95.4% in the validation and verification process. However, this study has gathered enough handwriting data from ET patients.

Zhao et al.^18^ developed a deep CNN based framework to identify calligraphy imitation (CI). The main aim of CNN is to detect the CI features and patterns available in handwritten signatures. Results show that the proposed CNN framework enhances verification accuracy by 96.8% and maximizes system performance. However, CNN-based methods need many handwriting samples from the writer.

Yang et al.^19^ proposed an unsupervised model-based handwriting posture prediction method. The proposed method achieves a high accuracy of 93.3% in posture prediction of 76.67%, improving the systems’ significance and effectiveness levels. However, the dimensionality reduction data acquired by executing the t-SNE method with the same parameters is distributed differently, necessitating further iterations of the chosen distribution map as the experimental data.

Al-Haija et al.^20^ designed a handwriting character recognition method based on the ShuffleNet CNN. CNN analyzed the datasets that are presented in the database. Compared with other methods, the proposed ShuffleNet CNN-based method maximizes the accuracy of 99.50% in character recognition. However, the sample size utilized in this study is small.

Ruiz et al.^21^ developed an offline handwritten signature verification method using a Siamese NN (SNN). The proposed method increases accuracy by 99.06% in the signature verification process, improving the systems’ efficiency. Limitation of SNN models is that they necessity huge amounts of labeled data for training, and accessible signature databases are usually small (concerning the no. of original signatures per writer).

Xue et al.^22^ introduced an attention-based two-pathway densely connected convolutional network (ATP-DenseNet) for gender identification of handwriting. A convolutional block attention module (CBAM) extracts the word features from signatures.Results show that the proposed ATP-DenseNet method achieves high accuracy by 66.3% in gender identification. Cropping the word pictures for ATP-DenseNet is difficult for papers with a lot of cursive writing, and the results are impacted by the cropping strategy used for the word images.

Hamdi et al.^23^ proposed a new data augmentation method for multi-lingual online handwriting recognition (OHR) systems. The geometric method is mostly used in the augmentation method that predicts the online handwriting trajectory (OHT). The proposed method improves the effectiveness of the systems by increasing accuracy by 97.2% in the recognition process. Some labels in the database contain a limited no. of samples.

Bouibed et al.^24^ developed a SVM based writer retrieval system for handwritten document images. SVM classifier is used here to classify the documents based on certain conditions and patterns. Results show that the proposed enhances the performance and feasibility of the systems by 96.76%. However, documents must be sorted from most to least similar to accomplish the writer’s recovery.

Alpar et al.^25^ introduced a new online signature verification framework using signature barcodes. The introduced framework maximizes the verification accuracy by 97.9%, improving the online systems’ efficiency and reliability. However, this method requires the selection of an optimal wavelet among many alternative features.

Maruyama et al.^26^ designed an intrapersonal parameter optimization method for offline handwritten signature augmentation and used in an automatic signature verification system (ASVS). The goal of the ASVS is to increase the accuracy of signature sample and predicts the writer’s variability and features. The proposed method improves the performance by 95.6% and the effectiveness range of ASVS. However, optimizing parameters may not produce more compact clusters in a feature space like the Critical carE Database for Advanced Research (CEDAR) dataset.

Zenati et al.^27^ presented a signature steganography document image system using beta elliptic modeling (SSDIS-BEM). A binary robust invariant scalable Keypoint (BRISK) detector is used here that detects the exact positions and identities of signatures. Compared with other methods, the proposed SSDIS-BEM maximizes accuracy by 84.38% in the signature verification process. However, lossy compression is not as stable since the discrete cosine transform (DCT) compression technique significantly alters the pixel intensities of embedded documents, making lossy compression less effective.

Wei et al.^28^ introduced an online handwritten signature verification based on the sound and vibration (SVSV) method for online systems. The SVSV method increases signature verification accuracy by 98.4%, improving online systems’ performance and efficiency. However, the robustness of the signature at various positions is considerably lower.

Cadola et al.^29^ proposed a collaborative learning-based teaching approach for forensic students for the learning process, which provides effective strategies to verify signatures. The proposed approach maximizes the overall efficiency of 93.4% of the learning process for forensic students. However, some characteristics remained problematic to forge. (e.g., loops or angles and commencements or terminations).

Houtinezhad et al.^30^ developed a feature extraction fusion (FEF) based writer-independent signature verification method. Canonical correlation analysis (CCA) is used here to analyze the discriminative features presented in a signature. The proposed method enhances the performance and reliability of systems by increasing accuracy by 86% in the verification process. Limitations include relying on a single reported visual representation and lacking further data for authentic and forged signatures.

Zhou et al.^31^ proposed the Dual-Fuzzy (DF-CNN) for handwritten image recognition. This study shows the calculation process, including estimates for forward propagation, backward propagation, and changing parameters. The DF-CNN’s optimization method is given so that its best results can be found. The DF-CNN and its optimization method are used to solve a real problem: recognizing writing numbers. The calculation process and the comparison show that the suggested new model and method are both feasible and beneficial. However, the sample images used in this study are small in numbers.

Ponce-Hernandez et al.^32^ suggested the Fuzzy Vault Scheme Based on Fixed-Length Templates for Dynamic Signature Verification. Fifteen global parts of the signature are used to make the models. The success of the suggested system is measured using three databases: a private collection of signatures and the public databases MCYT and BioSecure. The testing results show that the evaluation performance is higher than existing models. The high time inefficiency of this technique arises from the fact that it must assess several candidates’ polynomials for each authentication attempt.

Abdul-Haleem^33^ created an offline signature verification system that utilized a combination of local ridge characteristics and additional features derived by using the two-level Haar wavelet transformation. Each wavelet sub-band image is divided into overlapping blocks, local characteristics and wavelet energies retrieved from each block. For verification, the system’s FRR was 0.025% and its FAR was 0.03%. The varying choices of block lengths and overlapping ratios have a significant impact on the recognition rate.

According to the various researcher’s, handwriting is recognized by applying various neural networks and machine learning techniques. These techniques consume high computation time and face difficulties while identifying the different writing styles. Some methods require substantial data, challenges with dimensionality reduction or the need for optimal wavelet selection as listed in Table 1. Among all the literatures reviewed above the three existing methods such as SV-SNN^21^, AVN^16^, and SVSV^28^ are enclosed for comparison purpose. The research difficulties are overcome by applying the spatial variation-dependent verification scheme.

**Table 1:** Detailed analysis of existing related work.

Ref. paper	Main focus	Findings	Limitations
^16^	AVN for authenticating signatures	94% accuracy in verification and feature extraction	Slower performance with less variation
^17^	Transformer model for analysis of signatures	95.4% accuracy and 97.8% efficiency	Inadequate data about ET victims
^18^	CNN for identifying and detecting calligraphy	Verification accuracy of 96.8%	Many handwriting samples are necessary
^19^	Predicting handwriting position without supervision	93.3% posture prediction accuracy	Challenges of dimensionality reduction
^20^	ShuffleNet CNN in order to identify characters	99.50% accuracy in character recognition	Limited Sample size
^21^	Siamese neural network system for verifying signatures	99.06% accuracy in verification, less time and effort	Needs large labelled data
^22^	Gender identification using ATP-DenseNet	Gender identification accuracy of 66.3%	Cropping difficulties
^23^	Data enhancement for OHR systems	Geometric technique, 97.2% recognition accuracy	Limited samples in some labels
^24^	A writer retrieval system based on SVM	improved performance and feasibility by 96.76%	Signature documents must be sorted
^25^	Online signature verification with barcodes	97.9%verification accuracy with wavelet selection	Wavelet selection was optimal
^26^	Signature augmentation parameter optimisation	Parameter optimization enhanced performance by 95.6%	Compact clusters were not produced
^27^	BEM signature steganography	Verification accuracy of 84.38%, reduced computation time	Instability of lossy compression
^28^	For online systems, the SVSV technique was employed	Signature verification accuracy of 98.4%, sound and vibration	Robustness inconsistence in a variety of positions
^29^	Forensic students can benefit from collaborative learning	Learning efficiency of 93.4%, mistakes and complexity reduced	Some qualities are problematic
^30^	Fusion of feature extraction for writer-independent	CCA analysis, LBP features, and 86% verification accuracy	Visual depiction is limited
^31^	Handwritten image identification using dual-fuzzy CNN	Model and approach that is both feasible and useful	Limited samples
^32^	Dynamic Signature Fuzzy Vault Scheme	Improved evaluation performance and efficiency	Inefficient use of time
^33^	two-level Haar wavelet transformation	Improved recognition accuracy, FAR (0.030%) and FRR (0.025%)	Occurrence of overlap

### Proposed spatial variation-dependent verification (SVV) scheme

#### Problem statement

A person’s handwriting evolves and changes, making it a behavioral biometric. It requires cooperation between the brain’s motor effectors (the hands) and the body’s sensory organs (the eyes). The coordination of these systems enables humans to create intricate ink patterns and sequences. To create reliable writer identification systems, scientists have studied the behavioral side of writing styles, or “handwriting biometrics”. For decades, scientists have studied handwriting as a proxy for personality. Multiple disciplines have a common fascination with a person’s handwriting. Forensic scientists, psychologists, and palaeographers are all examples. Both the character style and the literary style vary considerably from one another. Authorship of a handwritten document may be determined by a procedure called handwriting identification. Three stages are involved in establishing authorship from the handwritten text: data collecting and preprocessing, feature extraction, and classification. Obtaining features that accurately represent the many types of handwriting is the primary challenge in handwriting recognition. Although several feature extraction methods have been shown in the research and put into practice for handwriting recognition, the literature does not provide enough information to fully analyze the significance of every given feature in handwriting recognition.

**Figure 1:**
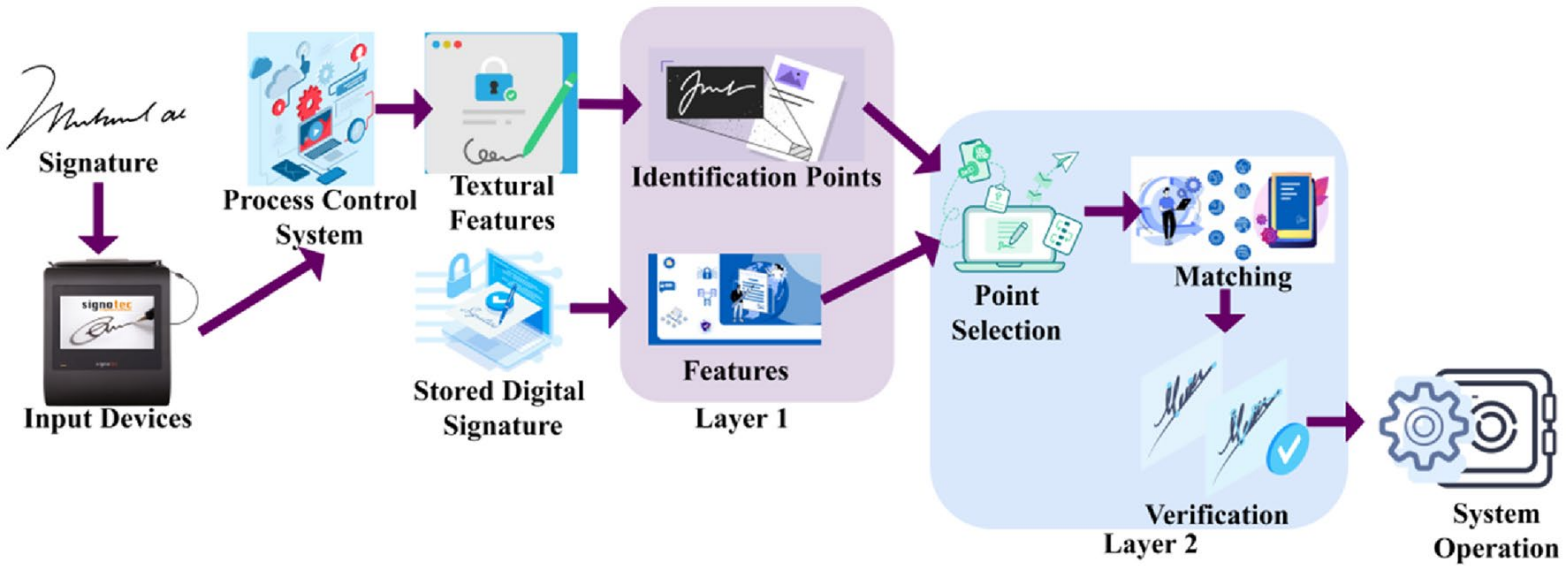
Proposed SVV scheme.

This study proposes a SVV scheme using TF. First, the pixel intensities used to identify identification points in handwritten and digital signatures are checked for accuracy. This distinguishing feature varies depending on the signature’s design, location, and texture. To confirm the matching of textural features, the selected spot is digitally signed and placed on a spatial map. The derived textural characteristics are used between two consecutive identification locations to avoid accumulating false positives. A CNN aids this layered analytical method. The first layer produces New Identification, while the second layer chooses the most optimal matching feature for intensity changes. The proposed SVV scheme defines the Identification and verification of handwriting signatures to ensure better textural feature extraction in centralized intelligent process control systems. The handwritten texts generally contain a unique writing style for each individual. Distribution of these handwritten signatures is used for defining writing style. The influencing textural features, such as pixel intensities and spatial variations, are detected by handwriting verification synchronized for identifying the writer’s sex and writing style. It ensures the handwritten and digital signatures for identification point detection. In a heterogeneous environment, the handwritten image ${H}^{image}$ is serving input through the device for recognition and verification, for writer identity $Wr$. Figure 1 presents the proposed scheme’s process.

The texture detection is modeled for the feature-matching process and the handwritten signatures are verified for heterogeneous writers in that the control system consists of female $F$, male $M,$ and other genders $O$. The handwritten and digital signatures are verified using two successive identification points based on a convolutional neural network. Identification points are distinguishing characteristics of an individual’s handwriting signatures. They are essential for validating and identifying between different authors. Detecting these different signals ensures signature verification accuracy and precision. Matching identifying points reduces $FP$, which contributes to more reliable findings.

Let $P$ represent the process control system consisting of $K\left({H}^{image}\right)$ handwritten signatures in the available control system for handwriting verification the input device ${in}^{d}$ generates a handwritten signature is expressed as:

1 $$\left.\begin{array}{c}K\left({{H}^{image}}^{1}\right)=SV\left[\Delta \left({F}_{1}\oplus {M}_{1}\oplus {O}_{1}\right)\right]\\ K\left({{H}^{image}}^{2}\right)=SV\left[\Delta \left({F}_{2}\oplus {M}_{2}\oplus {O}_{2}\right)\right]\\ \begin{array}{c}\vdots \\ K\left({{H}^{image}}^{P}\right)=SV\left[\Delta \left({F}_{N}\oplus {M}_{N}\oplus {O}_{N}\right)\right]\end{array}\end{array}\right\}.$$

Equation (1) computes $SV(.)$ as the spatial variations, $\Delta$ varying pixel intensities, $VT$ is the time for verifying the handwritten signature with input devices as per Eq. (2). The variable ${T}_{g}$ is the handwritten or digital signature generation time, ${T}_{i}$ is the overall time for identifying handwriting and ${T}_{m}$ is the identification point and feature matching time.

2 $$\left.\begin{array}{c}VT=\sum_{i=1}^{P}{T}_{g}-\left(1-\frac{{T}_{m}}{{T}_{i}}\right)\\ \forall N={H}^{image} \,or\, N<{H}^{image}\\ and\\ N\in verification\, of \,{H}^{image}\end{array}\right\}.$$

**Figure 2:**
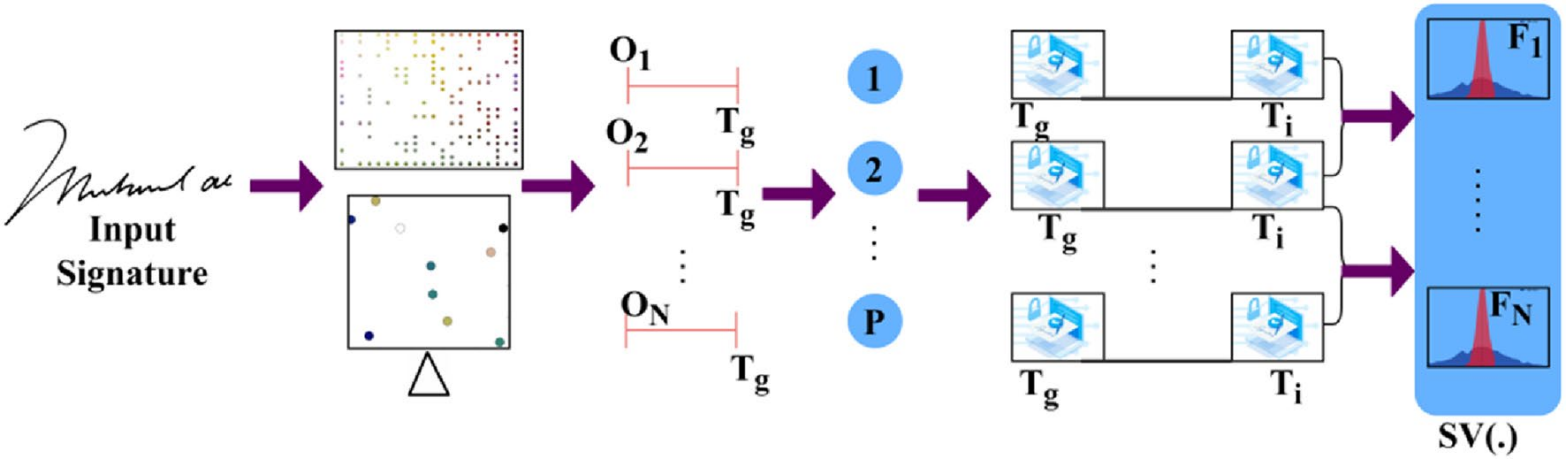
Spatial variation estimation.

$N<{H}^{image}$ is to satisfy all the digital and handwritten signatures from the input device verified at any time $VT$. This authentication controls the anonymous changes the hacker or any other person performs during the confidential process. The authentication process is a security featurre that ensures the reliability of the network and the information that is being processed by allowing only authorised and legitimate changes while detecting and mitigating unauthorised or malicious changes. This is extremely crucial in systems that use AI to maintain process correctness and reliability. The spatial variation estimation process is illustrated in Fig. 2.

The input is first segregated along $x$ and $y \forall \Delta \in {O}_{1}$ to ${O}_{N}$ occurs for ${T}_{g}$ alone $\in P$ provided $\Delta$ is high/low, depending on the observed ${T}_{g}$. ${F}_{1}$ to ${F}_{N}$ is validated for extracting the variations and the spatial lookups match the input with the stored ones. Consider the approximate total of $n=2500$ for the special textural feature of letters. For the digital copies of handwritten signatures using identification points, as in Eq. (3):

3 $$Wr=\left\{\begin{array}{l}Male, \quad if \,h\left(x\right)>0\\ Female,\quad if \,h\left(x\right)<0\\ Other \, gender, \quad if\, h\left(x\right)=0\end{array}.\right.$$

Equation (4), ${idf}_{p}\left(x\right)$ denotes identification point’s detection in a CNN for generating new:

4 $${idf}_{p}\left(x\right)=\sum_{i=1}^{n}{\alpha }_{i}{x}_{i}+FP.$$

Equation (5), ${\alpha }_{i}$ means stored digital signature, ${x}_{i}$ means extracted features, and $FP$ means false positive:

5 $${\alpha }_{i}={\text{log}}\frac{{b}^{i}\left(1-{a}^{i}\right)}{{a}^{i}\left(1-{b}^{i}\right)},$$

6 $$FP=\sum_{i=1}^{n}{\text{log}}\frac{{b}^{i}\left(1-{a}^{i}\right)}{{a}^{i}\left(1-{b}^{i}\right)}+\beta .$$

Equation (6) shows, ${b}^{i}=a\left({x}_{i}=1/{Wr}_{1}\right)$ indicates the $ith$ textural feature computation with a probability of a male writer ${b}^{i}\ne \left\{\mathrm{0,1}\right\}$; ${a}^{i}=b\left({x}_{i}=1/{Wr}_{2}\right)$ is the $ith$ textural feature computation with a probability of a female writer ${a}^{i}\ne \left\{\mathrm{0,1}\right\}$ and ${a}^{i}{b}^{i}=ab\left({x}_{i}=1/{Wr}_{3}\right)$ indicates the $ith$ textural feature computation with a probability of other gender writers ${a}^{i}{b}^{i}\ne \left\{\mathrm{0,1}\right\}$. If $\beta$ represents identifying special characters. The number of features of handwritten text $L$ is the serving inputs to the devices and the identification points in CNN are expressed as in Eq. (7):

7 $$\left.\begin{array}{c}\overline{h }\left(x\right)=\sum_{i=1}^{n}{\acute{\alpha} }_{l}{x}_{i}\\ where\\ {\acute{\alpha} }_{l}=\frac{{b}_{i}}{{a}_{i}},{a}_{i}\ne 0 \end{array}\right\}.$$

**Figure 3:**
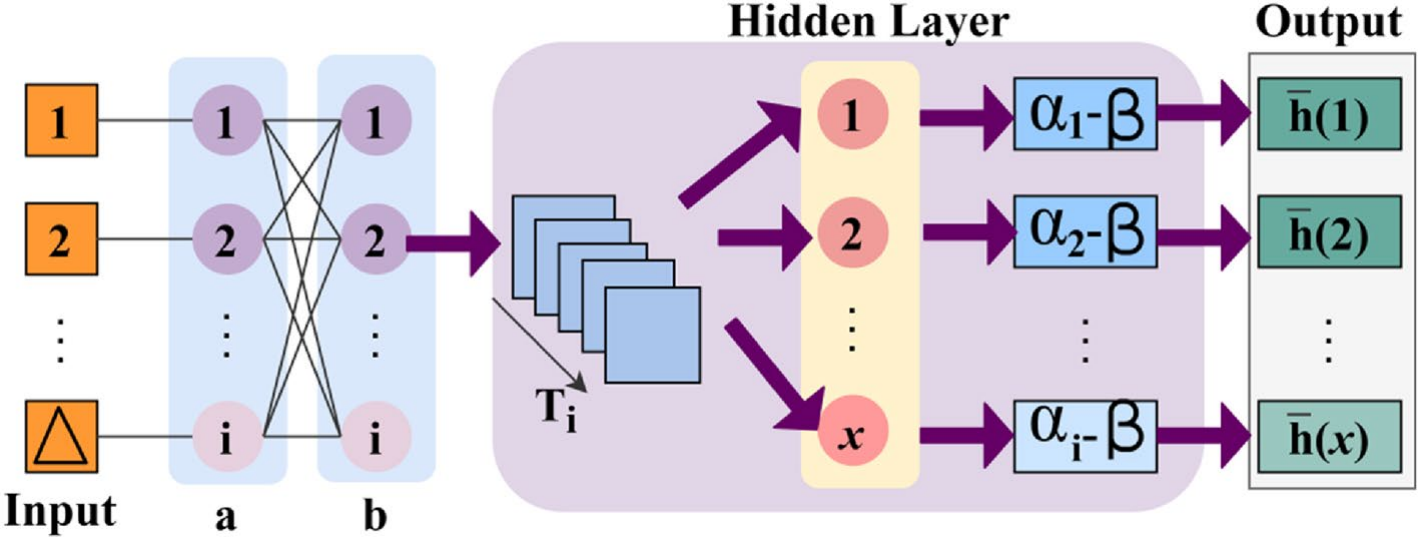
CNN process for identification points.

In this first layer, the generating new identification points and their varying features are analyzed using CNN with the already stored digital signature. The CNN process for identification points is presented in Fig. 3.

The input $\forall \Delta$ is validated for ${a}_{i}{b}_{i}$ across ${T}_{i}$; conceals ${T}_{g}$ and ${T}_{m}$ such that $SV\left(.\right)$ is detected. In the $SV(.)$ detection process, $x$ is the key factor for detecting identification points present as $\left({\alpha }_{i}+{x}_{i}\right)$ that causes $FP$. Therefore, the $\beta$ from different $i$ instances are validated for preventing $FP$ and generates $\overline{h }(x)$ output with precise identification points (Fig. 3). The detection of identification points are eligible to match textural feature with the $K\left({{H}^{image}}^{P}\right)$ depending on the authentication using $L$ as in Eq. (8):

8 $$\left.\begin{array}{c}L\left(Wr\right)={idf}_{P}[{N}^{s}|\left|K\left({{H}^{image}}^{P}\right)\right]\\ for\, all\\ \begin{array}{c}{N}^{s}\in N\le {H}^{image}\\ K\left({{H}^{image}}^{P}\right)\in {t}_{g}<VT\end{array}\end{array}\right\}.$$

The writers identify the identification point to verify the textural feature matching, if $N<{H}^{image}$ then ${\left({H}^{image} \right)}^{N}$. This process is distinct for the $N={H}^{image}$, $N>{H}^{image}$ and $N<{H}^{image}$ conditions, if $N<{H}^{image}$ is modeled as a layered analysis for identifying handwriting based on verification time is similar for all the writers irrespective of $N$ and $VT$. In essence, Case 1 and Case 2 are utilized to categorize and address various operating circumstances within the proposed SVV-TF. They aid in the definition of how the system adjusts and manages identification points for proper verification. Case 1 describes a situation in which the number of created identification points equals the count of handwritten signatures. It’s a circumstance in which each generated point may be identical using a specific signature, resulting in a simple and effective verification method. Case 2 refers to situations in which the total quantity of computed identification points is smaller compared to the count of handwritten signatures. This situation is further subdivided into scenarios in which the number of points in respect to the total amount of signatures is even or odd. Specific algorithms are used to manage these cases, ensuring reliable verification despite the fact the total quantity of elements and signature differ.

*Case 1* The ${H}^{image}$ count is the same as the no. of generated identification points.

*Analysis 1* This is the ideal case for all the writers where the efficiency of the generated identification point is not matched $K\left({{H}^{image}}^{P}\right)$ then completely cut down the iterated verification. Here, the first level is responsible for processing and generating new identification points based on $N$ or ${H}^{image}$. ${F}_{F}$ represents the false factor identified by the input device that serves as the root of the second layer. The notion of false factor most likely refers to inaccuracies or erroneous information added throughout the identification as well as verification procedure. The ${F}_{F}$ denotes situations in which the system wrongly recognizes an individual or fails to identify their identification points. Reduced ${F}_{F}$ is critical for increasing handwriting recognition system accuracy and dependability.

The output of ${F}_{F}$ as $\left\{K\left({{H}^{image}}^{1}\right), K\left({{H}^{image}}^{2}\right),\dots K\left({{H}^{image}}^{P}\right)\right\}$ is assigned to the individual writers. In the textural feature extraction, $\forall N={H}^{image}$, the pursuing writer’s handwriting is matched with the already stored signatures in the following manner shown in Eq. (9):

9 $$\left.\begin{array}{c}\left[K\left({{H}^{image}}^{P}\right)\times {F}_{F}\left|{Idf}_{P}\right|\right]\Vert \left[L\left(Wr\right)\times {\alpha }_{i}\times \delta |{Idf}_{P}\right]\Vert \left[L\left(UE\right)\times {L}_{P}|{Idf}_{P}\right]\\ where, \\ \delta =\left[{a}_{i}^{{F}_{F}}\oplus \left({b}_{i}^{{F}_{F}}\frac{1}{\beta } \left|{Idf}_{P}\right|\right)\right]\end{array}\right\},$$

where $\delta$ is the generated point selection based on the writing style of the writer, is identified without false factor and matches its spatial variations and pixel intensities ${a}_{i}^{{F}_{F}}$ and ${b}_{i}^{{F}_{F}}$ with stored signatures.

*Case 2* The ${H}^{image}$ the count is less than the identification point generated (i.e.) $N<{H}^{image}$.

**Figure 4:**
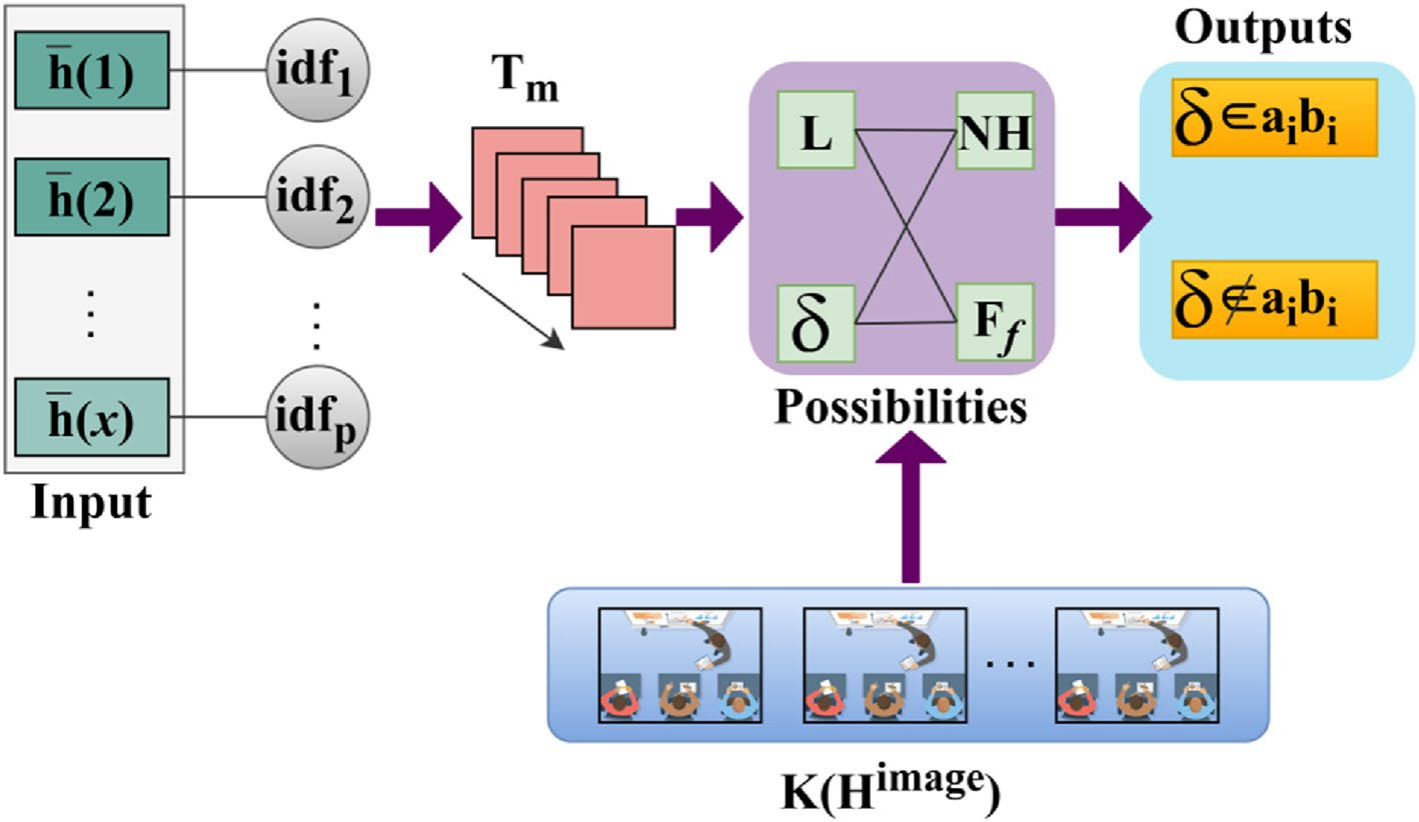
Point selection using CNN.

*Analysis 2* The role of digital or handwritten signatures and Identification of the writer makes it reliable for reducing the chances of a security vulnerability without maximizing the computation complexity. Therefore, $N<{H}^{image}$ such that $\frac{{H}^{image}}{N}=even \,or\, odd$ for which the selection point satisfies maximum matching, point selection considering the above cases through the CNN is presented in Fig. 4.

The first layer’s input (i.e.) $\overline{h }(1)$ to $\overline{h }(x)$ is fed as input for $id{f}_{p}$ provided the analysis is performed in ${T}_{m}$ alone. This reduces the actual time required for preventing $(N-H)$ occurrence $\forall {F}_{f}$. Therefore with the available $\Delta$ and $K\left({H}^{image}\right)$ the possibilities of $L \& \delta$ are validated for $\left(N-H\right)$ and ${F}_{f}$. In this possibility, $\delta \in {a}_{i}{b}_{i}$ and $\delta \notin {a}_{i}{b}_{i}$ is classified as output. The selection points $\forall \delta \in {a}_{i}{b}_{i}$ are used for confining $SV\left(.\right)$ (Fig. 4). In this case $\frac{{H}^{image}}{N}=odd/even$, after the sequential feature matching waiting for the handwriting signature verification $(VR)$ and then performing system operation. This process is estimated as $[(VR-L)/|{F}_{f}|+1]$ and $|FP|$ is the maximum false rate occurrence identification in the second layer. Here, $\forall 1\le N<{H}^{image}$, the maximum matching feature for varying intensity is expressed as:

10 $$\left.\begin{array}{c}\left[L\left(Wr\right).FP\right]\times \left[\left|{Idf}_{P}\right|\frac{1}{N-{H}^{image}}||\oplus {b}_{Ni}\right]=\left[\left(\left|{Idf}_{P}\right|\times {L}_{N-{H}^{image}}\right)\oplus {a}_{Ni}\right]\\ Now,\, the\, point\, selection\, count \,is \,reduced\, to \,varying\, intensity\,{ v}_{int}\\ where\, S=\left(\frac{\left(V-L\right)}{\left|H\right|+1}\right)\\ and\\ \left[{L}_{N-{H}^{image}}.FP\right]\left[\left|{{Idf}_{P}}_{N-{H}^{image}}\right|\frac{1}{N-{H}^{image}}\oplus {b}_{N-{ v}_{int}}\right]=\left[\left|{{Idf}_{P}}_{N-{H}^{image}}\right|\times { v}_{int}\oplus {a}_{N-{ v}_{int}}\right] \end{array}\right\}.$$

Equation (10) estimates the precise digital or handwriting signature verification based on the sequence of textural feature extraction whereas the identification point and texture feature does not match $\frac{{H}^{image}}{N}=odd$. On the other hand, the signature verification is different in this case $\frac{{H}^{image}}{N}=even$ (i.e.) $Wr-2({H}^{image}-N)$ is the considered instance for verification expressed as:

11 $$\left.\begin{array}{c}\left[L\left(Wr\right). FP\right]\times [{v}_{int}\frac{1}{Wr}|\left|\oplus {b}_{Ni}\right]=\left[\left|{Idf}_{P}\right|L\oplus {a}_{Ni}\oplus {b}_{Ni}\right]\\ \left[L\left(Wr\right).{F}_{f}\right]\times [{v}_{int}\frac{1}{Wr}|\left|\oplus {b}_{Ni}\right]=\left[\left|{v}_{int}\right|L\oplus {a}_{Ni}\oplus {b}_{Ni}\right]\end{array}\right\}.$$

**Figure 5:**
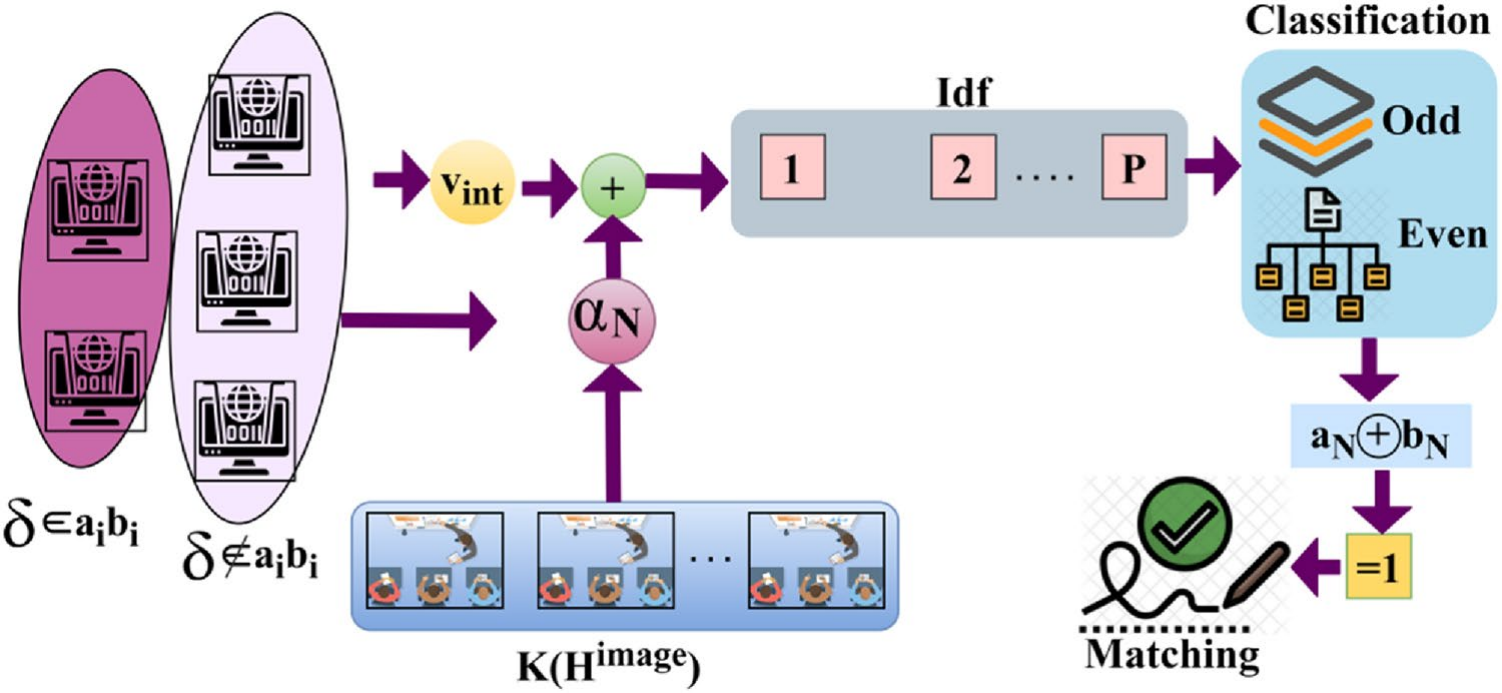
Matching process.

Equation (11) indicates the minimum possible computation required for handwritten Identification and verification. In this series, the handwritten signature verification process time as varying by the above condition ${T}_{g}$ and $VT$ instance. The matching process for signature verification is illustrated in Fig. 5.

**Figure 6:**
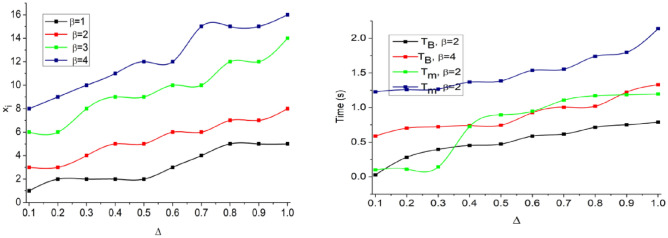
$${T}_{g}$$, $${T}_{m}$$, and $${x}_{i}$$ analysis.

The CNN-classified outputs are used for ${v}_{int}$ differentiation from $K\left({H}^{image}\right)$ such that any of ${V}_{int}\oplus {\alpha }_{N}$ achieves $P\in Idf$. Depending on the availability, the even/ odd classification is observed from which ${a}_{N}\oplus {b}_{N}$ is performed. In the above process is the maximum (i.e.) ${a}^{i}{b}^{i}=1$, then matching is successful (Fig. 5). In Fig. 6, the analysis of ${T}_{g}$ and ${T}_{m}$, and ${x}_{i}$ for the varying $\Delta$ is presented.

**Figure 7:**
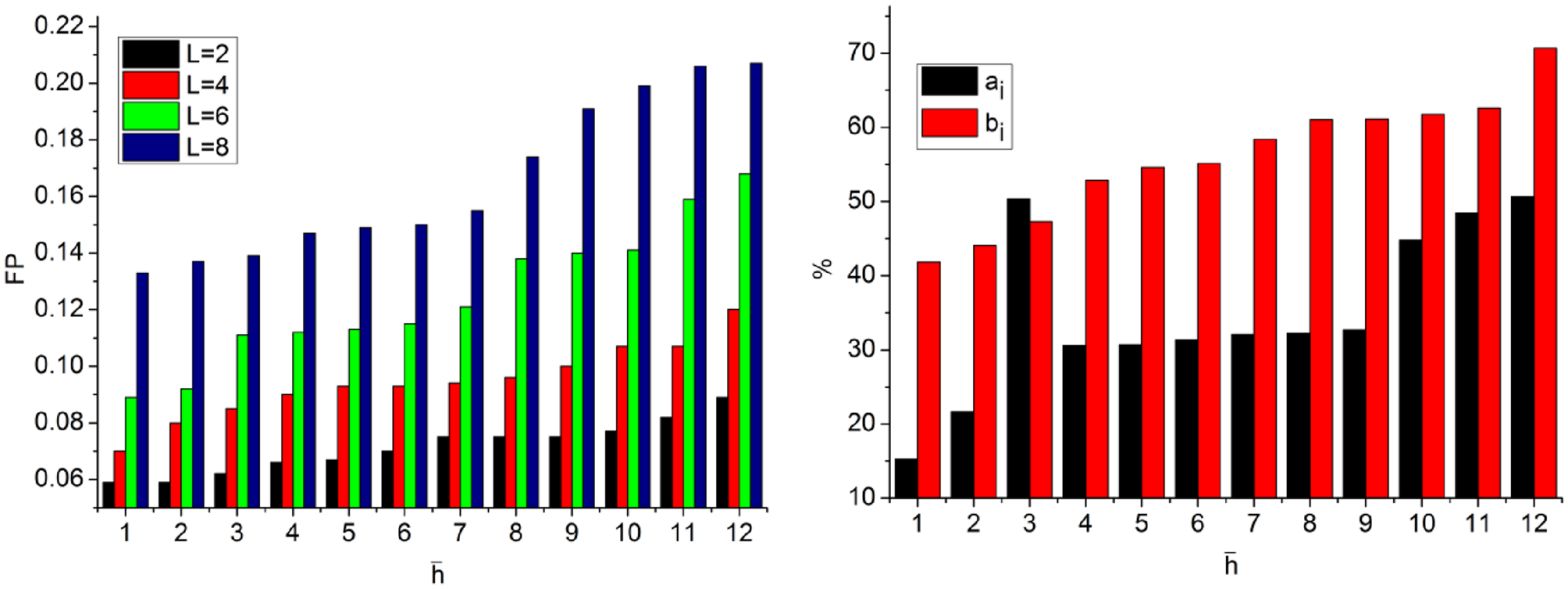
Analysis of $$FP, {a}_{i},\text{ and }{b}_{i}$$.

In the proposed scheme the ${T}_{g}$ and ${T}_{m}$ demands are variable depending on the $\beta$ occurrence. If $\beta$ occurrence is high, then $\left(N-H\right)$ becomes invariable such that $\overline{h }(x)$ increases. Therefore the $SV(.)$ is suppressed under controlled CNN layers. Precisely the first layer denies the $FP$ due to ${a}^{i}{b}^{i}\notin 1$ and hence ${T}_{g}$ is restricted then ${T}_{m}$. The ${x}_{i}$ increases with the $\beta$ for which $\delta$ and matching are precise. Based in the available $\Delta$ and $SV(.)$ classification, the ${\alpha }_{i}$ is distributed. The distributions are classified for $(N-H)$ and ${F}_{f}$ such that either of $\delta \left(\in {a}_{i}{b}_{i} or \notin {a}_{i}{b}_{i}\right)$ is the output. If the output is an $FP$, then ${x}_{i}$ increases and therefore, CNN’s layer 1 process is repeated. An analysis of $FP, {a}_{i}$ and ${b}_{i}$ for the varying $\overline{h }$ is presented in Fig. 7.

**Figure 8:**
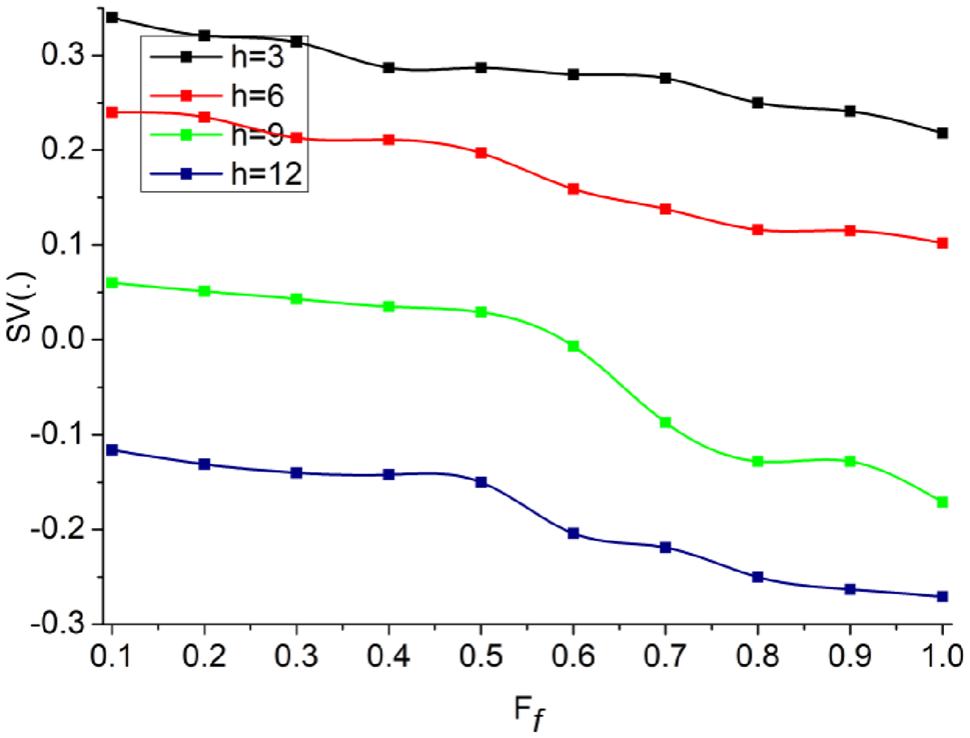
Analysis of $$SV(.)$$.

The analysis of $FP$ varies with $L$ as the $\delta \in {a}_{i}{b}_{i}>\delta \notin {a}_{i}{b}_{i}$. In this process, $\beta$ are omitted to satisfying ${a}_{i}{b}_{i}=1 \forall {a}_{N}\oplus {b}_{N}=1$. Therefore as $FP$ increases, the $L$ increases for confining them in consecutive iterations. As the iterations from layer 2 to layer 1 of the CNN are confined (repeated) the ${a}_{i}>{b}_{i}$ occurs (randomly), else the variations are less such that ${a}_{i}<{b}_{i}$ is the actual output (Refer to Fig. 7). The analysis of $SV(.)$ for varying ${F}_{f}$ and $\overline{h }$ is presented in Fig. 8.

The proposed scheme identifies $FP \forall {\alpha }_{i}\notin \left\{\mathrm{0,1}\right\}$ such that $SV$ occurs. This is due to the $\overline{h }$ occurrence, and therefore, new $L$ is required for confining $FP$. Therefore the considered $VT$ is used for confining $SV(.)$ for ${L}_{N-H}$ and ${F}_{f}$ variations. Therefore the considered intervals of ${T}_{i}$ (without ${T}_{m}$) is used for preventing $FP$ that does not require $SV\left(.\right)$ balancing (Fig. 8).

## Performance analysis

Imagery data from Ref.^34^ and the Handwritten Hebrew Gender Dataset^35^. In this dataset, genuine and fraud signatures are classified in 42 directories providing 504 testing inputs. The training set is randomly obtained from 128 directories containing 8 to 24 images. With this input, 16 textural features and a (0.1–1) intensity range is varied for analysing accuracy, precision, feature detection, false positives, and verification time. In the comparative study, the methods SV-SNN^21^, AVN^16^, and SVSV^28^ are enclosed with the proposed SVV-TF scheme. Sample input and output representing the key processes of the above discussion are tabulated in Tables 2 and 3. The signatures that are used for the analysis are from the authors.

**Table 2:**
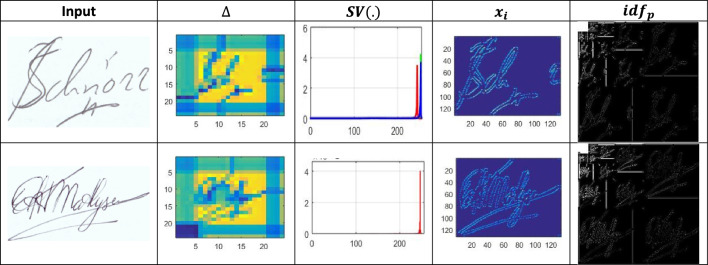
Identification points.

**Table 3:**
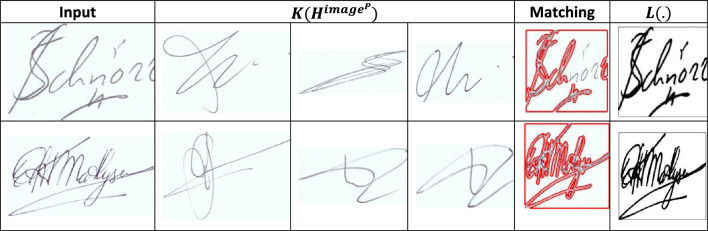
Matching and verification.

### Accuracy

**Figure 9:**
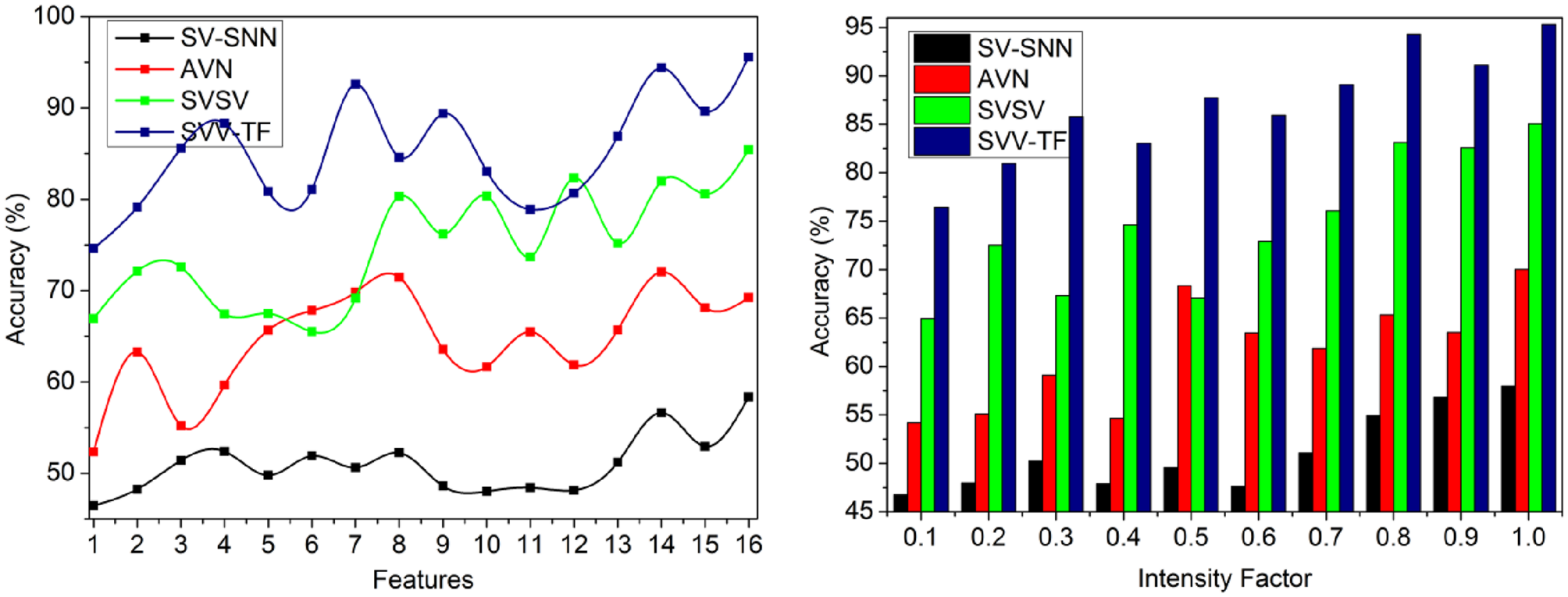
Accuracy analysis.

In Fig. 9, the term “intensity factor” most likely refers to the varied pixel intensities or brightness levels in a handwriting or digital signature. These changes in the intensity of pixels are inspected and analyzed within the framework of the process of identifying and confirming the handwriting, helping to recognize various textural aspects. The intensity factor may have an effect on the handwriting recognition system’s accuracy and precision. The spatial variation in handwritten signatures is identified with $\sum_{i=1}^{P}{T}_{g}-\left(1-\frac{{T}_{m}}{{T}_{i}}\right)$, the pixel intensities of the signature texture and outputs in point selection.

### Precision

**Figure 10:**
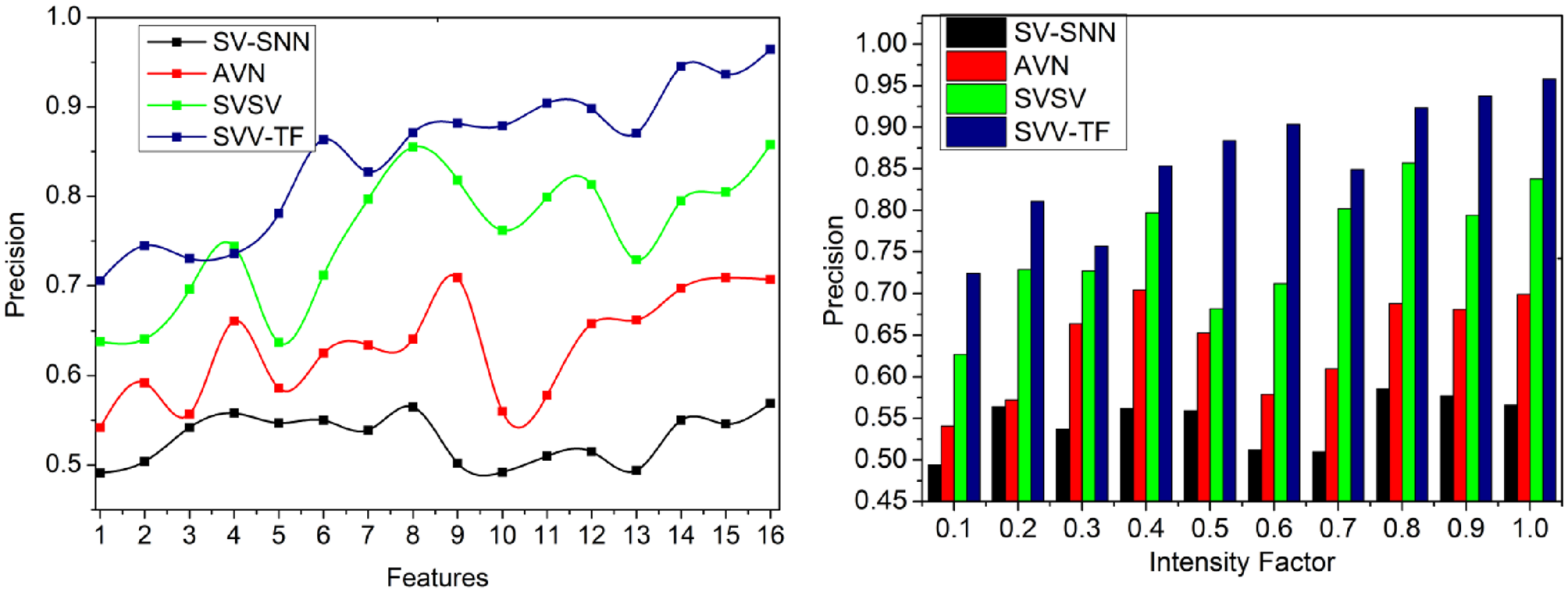
Precision analysis.

The variations in pixel intensities are identified for verifying the textural feature matching by performing the precise system operation based on the given handwritten signatures for identifying the difference between the stored digital signature and the current signature. In the first layer, the identification point is detected for recognizing the acute writer, and its writing style is deployed for identifying the spatial variations represented in Fig. 10. Therefore, the first and second levels are analyzed for accurate handwriting verification due to the surface and writing object changes being high precision for verifying the textural feature matching.

### Texture detection

**Figure 11:**
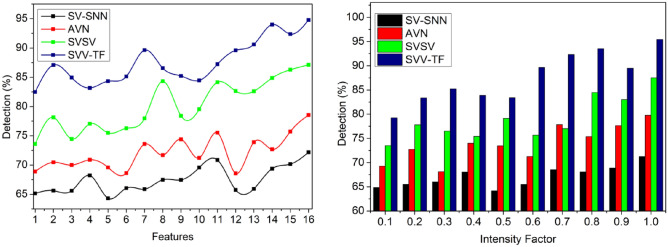
Texture detection analysis.

The textural features are analyzed and matched to improve handwriting signature quality for precise Identification; in this scheme, we detect the spatial variations and different pixel intensities based on the identification point illustrated in Fig. 11. Such that $N<{H}^{image}$ is to satisfy all the digital and handwritten signatures from the input device that can be verified at any time $VT,$ and a false positive occurs due to identifying spatial variations and pixel intensities in the pursuing signature.

### False positives

**Figure 12:**
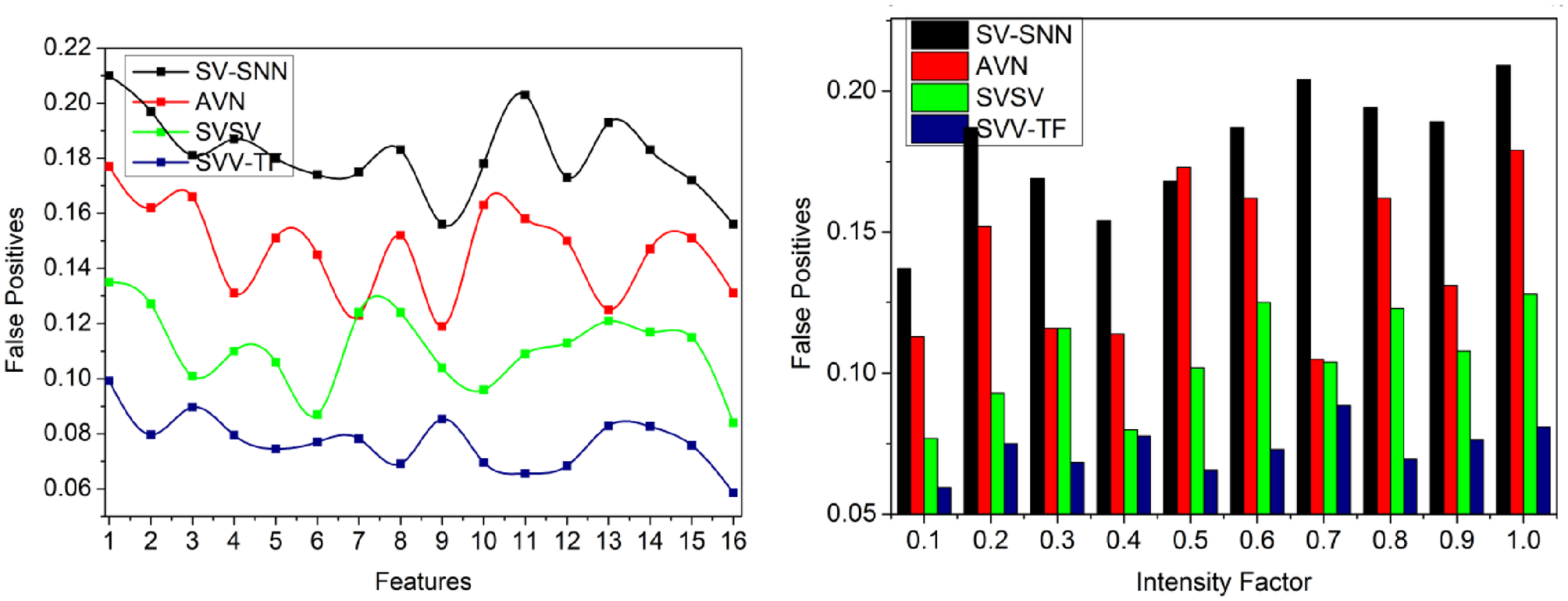
False positives analysis.

This proposed scheme detects the variations in pixel intensities using the textural feature extraction performed in the given input signature to prevent false factors at different time intervals. The verification of signature and identification point for the individuals from the texture feature matching output and then $N={H}^{image}$, $N>{H}^{image}$ and $N<{H}^{image}$ is computed using precise spatial variation and pixel intensity identification for time requirements. For instance, it achieves fewer false positives, as presented in Fig. 12.

### Verification time

**Figure 13:**
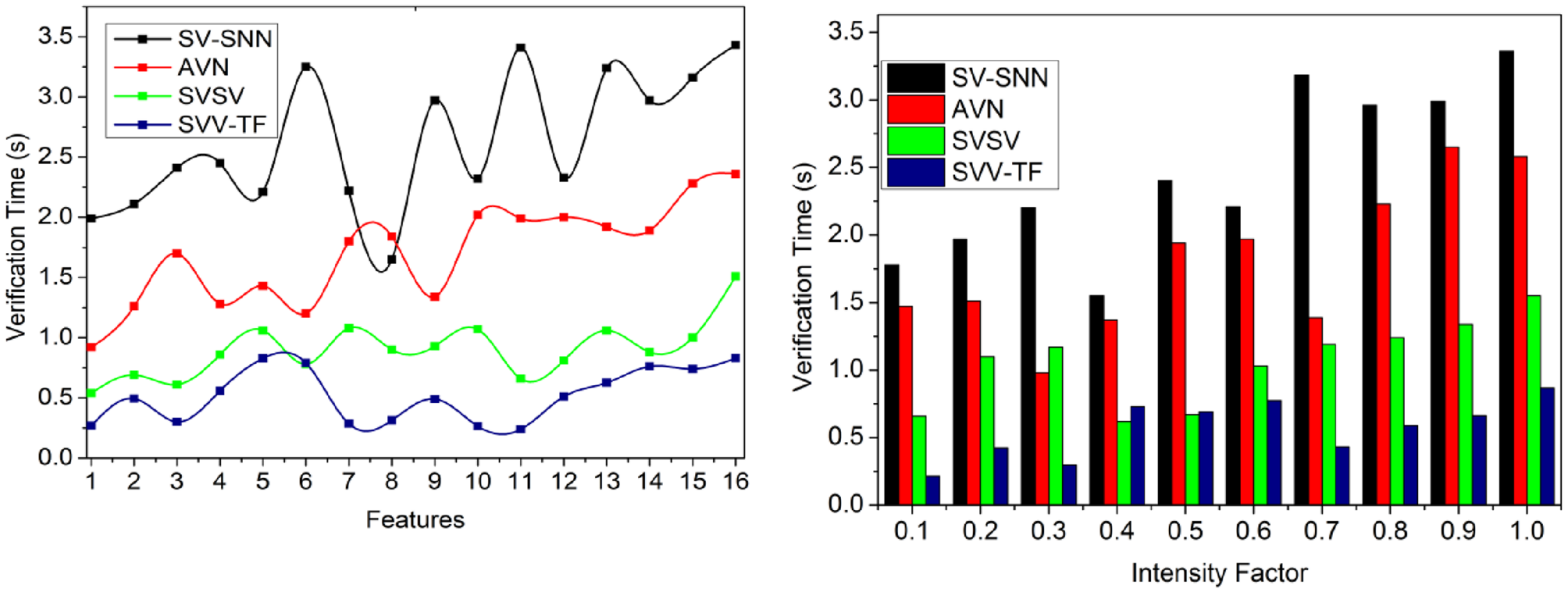
Verification time analysis.

It helps to satisfy less verification time for the pixel intensities and feature matching process compared to the other factors, as represented in Fig. 13. The pixel intensities are recurrently analyzed to match the process control system’s textural features and identification points. Based on the CNN, generating new identification points is performed to select the maximum matching feature for varying pixel intensity and analyzed for improving identification point detection. The handwriting verification process is similar for all the writers irrespective of $N$ and $VT$.

### False acceptance rate (FAR)

In biometrics and authenticating systems, the false acceptance rate metric (FAR) is often calculated to assess the rate at which the system wrongly accepts an impostor’s effort as a valid user. The total number of incorrect acceptances or signature matches called the total number of instances the system accepted an impostor’s exertion wrongly. The overall number of fraudulent efforts as the entire number of fraudulent tries.

12 $$FAR=\frac{no. \, of \, false \, acceptances}{Total \, no. \, of \, unauthorized \, attempts}\times 100\%.$$

**Figure 14:**
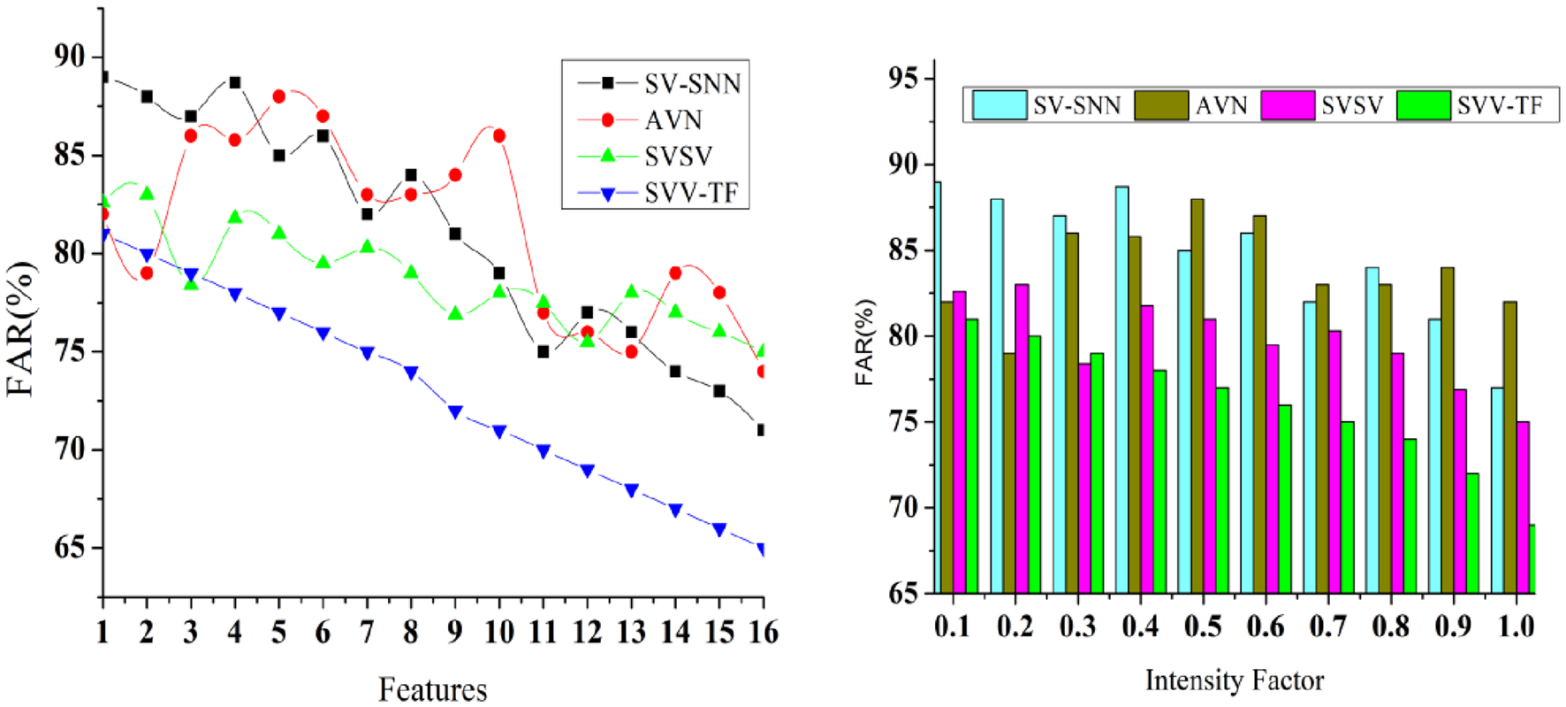
FAR analysis.

A high FAR in Fig. 14 implies that the system is accepting an unusually large number of unauthorised or fraudulent attempts is calculated using Eq. (12).

### False rejection rate (FRR) analysis

**Figure 15:**
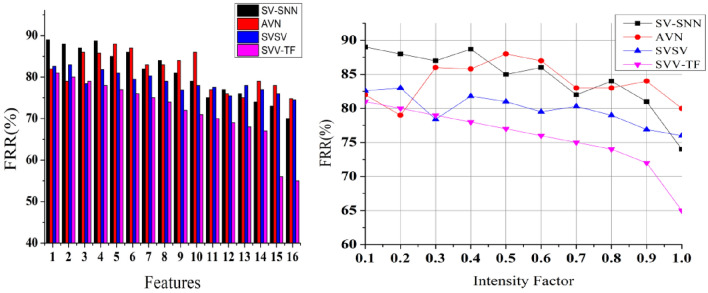
FRR analysis.

The FRR is an important indicator that indicates the system’s ability to accept valid signatures accurately. A minimal FRR is beneficial since it suggests that valid users are rarely rejected by the system.If the system wrongly rejects a real signature, it is considered a false rejection. A high FRR in Fig. 15, implies that the system is wrongly refusing many legitimate signatures, thus can be aggravating for users using Eq. (13). It is critical to evaluate and analyse FRR on a regular basis to guarantee that the signature authentication method provides an optimal user experience while preserving security.

13 $$FAR=\frac{no. \, of \, false \, rejections}{Total \, no. \, of \, genuine \, signatures}\times 100\%.$$

### Equal error rate (EER)

**Figure 16:**
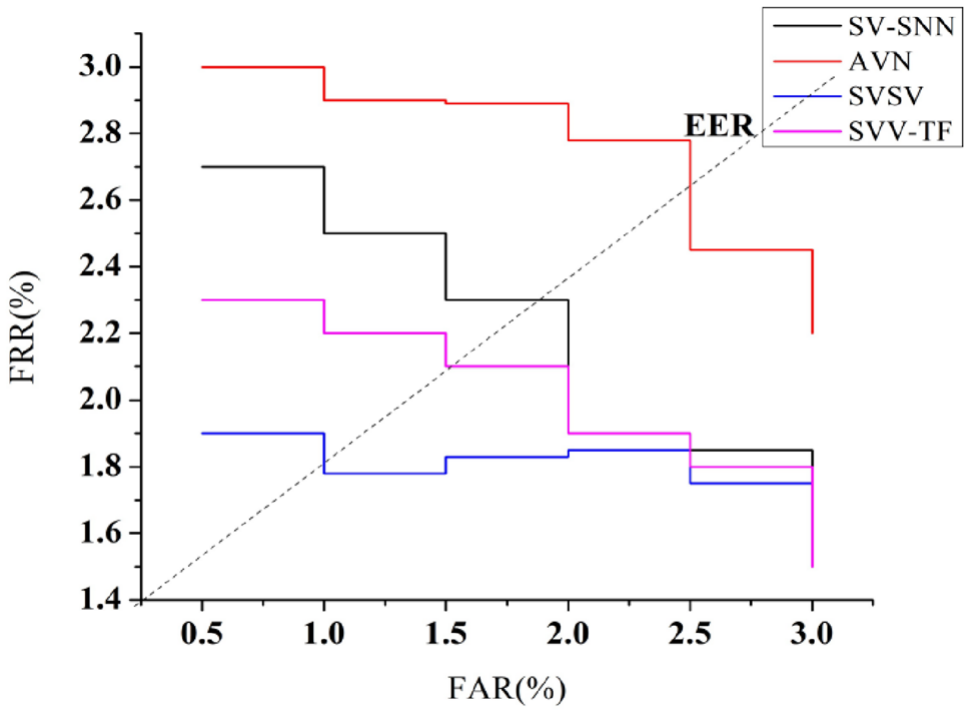
EER analysis.

The EER depicted in Fig. 16 is the region on the receiver operating characteristic curve in which the FAR and FRR are identical. The EER is a significant indicator since it reflects the operational point where the system’s efficiency strikes a balance between the danger of accepting an impostor wrongly and the risk of denying a legitimate user improperly. The EER is the ROC curve point at which FAR equals FRR.A lesser EER reflects a greater degree of precision system alongside fewer errors.

The above discussion of the comparative analysis is briefed in Tables 4 and 5, respectively, for features and intensity factors.

**Table 4:** Comparative analysis (features).

Metrics	SV-SNN	AVN	SVSV	SVV-TF
Accuracy (%)	58.37	69.25	85.42	95.587
Precision (%)	0.569	0.707	0.858	0.9644
Detection (%)	72.18	78.55	87.12	94.785
False positives(%)	0.156	0.131	0.084	0.0586
Verification time (s)	3.43	2.36	1.51	0.831
FAR(%)	71	74	75	65
FRR(%)	70	74.8	74.5	55

**Table 5:** Comparative analysis (intensity factor).

Metrics	SV-SNN	AVN	SVSV	SVV-TF
Accuracy (%)	57.98	70.03	85.04	95.301
Precision (%)	0.566	0.699	0.838	0.9579
Detection (%)	71.26	79.79	87.51	95.443
False positives(%)	0.209	0.179	0.128	0.081
Verification time (s)	3.36	2.58	1.55	0.869
FAR(%)	77	82	75	69
FRR(%)	74	80	76	65

#### Improvements

The metrics accuracy, precision, and detection are leveraged by 12.29%, 12.65%, and 15.5% in order. The metrics false positives and verification time are less than 13.01% and 10.99%. The accuracy, measures the overall correctness of the predictions made by each model. SVV-TF demonstrates the highest accuracy at 95.587%, indicating that it has the highest rate of correct predictions. Precision, the next metric, reflects the ratio of true positives to the combined total of true and false positives. SVV-TF also excels with a precision of 0.9644%, indicating that it has the lowest rate of false positives among all the models. Detection rate, evaluates the models’ ability to identify instances of interest accurately. SVV-TF leads with a detection rate of 94.785%, signifying its proficiency in correctly detecting the relevant instances. False positives, represent instances incorrectly predicted as positive. SVV-TF exhibits the lowest false positive rate at 0.0586%, indicating that it makes the fewest mistakes. The verification time measures how long each model takes to perform its task. SVV-TF is the quickest, taking only 0.831 s.

#### Improvements

The metrics accuracy, precision, and detection are leveraged by 12.14%, 12.85%, and 15.92% in order. The metrics false positives and verification time are less than 9.1% and 10.85%. SVV-TF makes accurate predictions more consistently than the other models. Precision, another crucial metric, signifies the ratio of true positives to the combined total of true and false positives. SVV-TF takes the lead with a precision of 0.9579%, highlighting its exceptional ability to minimize false positives. Detection rate, assesses the models’ proficiency in identifying instances of interest. Here, SVV-TF demonstrates remarkable performance with a detection rate of 95.443%, indicating its superior capacity to detect relevant instances accurately. False positives, representing instances incorrectly predicted as positive, are kept impressively low by SVV-TF at 0.081%. This further underscores its precision and effectiveness in making accurate predictions. The verification time measures each model’s time to perform its task. In this regard, SVV-TF is the quickest, requiring only 0.869 s.

## Conclusion

Handwriting Writers or writing Identification is figuring out who wrote a paper by analysing handwriting, text, and images. It has shown promise in many areas, such as digital forensics, crime investigations, finding out who wrote experienced papers, etc. It’s difficult to determine who wrote the text when the image is complicated, especially when there are different types of handwriting. The data are collected from the signature verification Kaggle^34^ and the handwritten gender dataset^35^. Offline signature verification or biometric signature verification works with scanned signatures, and online signature verification works with videos of the writing process. Compared to classical biometric-based handwritten signature identification is less accurate, and security issues will arise.

The proposed artificial intelligence and textural features attain high accuracy in handwriting identification. If the pixel intensities do not match the available textural features, the second layer is repeated from the new spatial variation for new pattern recognition. It enhances the accuracy of the varying feature inputs and training images. The metrics accuracy, precision, and detection are leveraged by 12.29%, 12.65%, and 15.5%. The metrics false positives and verification time are less than 13.01% and 10.99%. To guide future research, several promising avenues are suggested. These include extending the model’s capabilities to handle multiple languages, exploring advanced feature extraction methods, and incorporating temporal aspects of handwriting.

Additionally, there’s a recommendation to investigate online handwriting recognition and develop techniques for detecting forged or fraudulent handwriting. Transfer learning and domain adaptation techniques could be explored to adapt the model to different handwriting styles. Efficiency and scalability for real-world deployment, as well as considering security and privacy concerns, are emphasized. Lastly, establishing standardized benchmarks and evaluation metrics would facilitate fair comparisons between different handwriting identification and verification approaches. These directions hold the potential for significant application advancements related to authentication, security, and accessibility.

## Data Availability

All data generated or analysed during this study are included in this published article.
